# Biochar from Waste Vineyard Pruning as a CO_2_ Sorbent in Pressure Swing Adsorption: Experimental and Modeling Study

**DOI:** 10.1021/acsomega.5c00513

**Published:** 2025-06-11

**Authors:** Daniel Mammarella, Katia Gallucci, Andrea Di Giuliano

**Affiliations:** Department of Industrial and Information Engineering and Economics, 9303 University of L’Aquila, Piazzale E. Pontieri 1, Loc. Monteluco di Roio, 67100 L’Aquila, AQ, Italy

## Abstract

Vineyard pruning is an agro-industrial waste largely available in Italy. In this work, biochar from vineyard pruning pellets (previously produced by pyro-gasification at two Equivalence Ratios (*ER*)) was chemically activated and tested as a CO_2_ sorbent by pressure swing adsorption (PSA), at room temperature and 5, 7, or 9 bar_a_ (*P*). As-received and activated biochar samples showed CO_2_-sorption capacities (*Y*) comparable to or even higher than those of reference sorbents, emerging as good candidates for future PSA scale-up studies. A 2^3^ factorial design was adopted to study the dependency of measured *Y* on factors *P*, *ER*, and “activation”. Empirical linear regression models and Langmuir adsorption isotherms were obtained from *Y* experimental data. The extrapolation of the empirical model gave *Y* values with admissible deviations (lower than ± 5%) from the Langmuir isotherm in the range of 4–11 bar_a_. The proposed model is a good predictive tool for future PSA scale-up studies.

## Graphical abstract

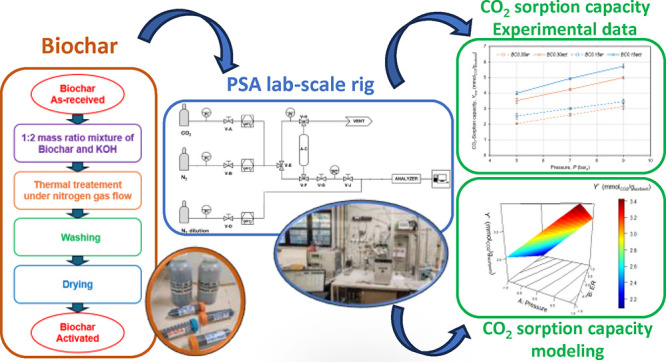

## 1 Introduction

The anthropogenic emissions of CO_2_ and related climate-change issues have attracted worldwide great attention and concern,^1,2^ for example, after the Paris Agreement of 2015.

CO_2_ emissions contribute by over 60% to global warming.^3–6^ The CO_2_ atmospheric concentration in 1958 was 315 ppm and reached 419.3 ppm on average in 2023.^7–9^ In 2023, CO_2_ emissions reached their new all-time maximum of 37.4 Gt.^10^ In 2022, the electricity and heat generation sectors marked the largest absolute sectoral increase in CO_2_ emissions, reaching the historical maximum of 14.6 Gt emitted.^11^

Solutions to control CO_2_ in the atmosphere (capture, storage, and reuse) have become a centerpiece of national and international policies to contain climate change.^1,12^ Following the Paris Agreement and subsequent policies, countries have committed to containing global temperature rise to +2 °C above preindustrial levels, preferably limiting it to +1.5 °C. Therefore, the development of efficient technologies for CO_2_ capture is essential to mitigate the impact of CO_2_ emissions on global warming, for example, in European Union with the ambitious goal of net zero emissions by 2050.^10,13,14^

The main technologies used for CO_2_ capture and separation include physical absorption,^15–17^ chemical absorption,^18–20^ adsorption,^21–23^ and membranes.^24–26^ Absorption and adsorption have been largely used and investigated in research.^14^ High investment costs and high thermal requirements for regeneration are the main hindering factors of absorption;^27^ furthermore, the use of chemical solvents leads to potentially significant environmental impacts.^18^ On the other hand, technologies based on physical adsorption, such as pressure-swing adsorption (PSA), are interesting candidates for a more sustainable separation and capture of CO_2_ at the industrial scale: PSA does not require heat and complex chemicals and is relatively cheap, easily scalable and compact.^27^ In this technology, pressure is a fundamental process parameter that characterizes both process performance and energy consumption. The operating PSA pressure is highly variable and depends on the specific application and gas mixture to be treated. Generally, a pressure range from slightly above atmospheric pressure to around 30–40 bar is used.^28,29^ Higher operating pressures can increase the adsorbed quantities of species to be separated as well as energy consumption due to fluid compression. For these reasons, it is essential to find a balance that ensures acceptable adsorption performance while keeping reasonably low energy consumption compatible with the coupling of PSA with processes at low pressure such as anaerobic digestion. As a result, several studies in the literature focus on material performance at pressures below 10–15 bar.^30–32^ Adsorption techniques for CO_2_ capture involve the use of solid sorbent materials, which are usually porous materials with high surface areas, maximized for effective CO_2_ capture.^14^ Activated carbon,^33^ zeolites,^34^ or carbon molecular sieves^35^ are some solid materials commonly used for CO_2_ capture by adsorption.

Along with the principles of circular economy,^36^ waste-derived solid sorbents can give added value to PSA in terms of sustainability. Biochar is obtained from thermochemical valorization of biomass and waste materials,^37–40^ usually by slow pyrolysis, or by fast pyrolysis, gasification, and hydrothermal carbonization;^41,42,43^ all these techniques involve an equivalence ratio (*ER*) lower than 1 (*ER* is the ratio between actual fed oxygen and stoichiometric oxygen for complete combustion). Recent usages of biochar have included exhaust gas purification, metallurgy, soil conditioning, animal husbandry, building, medical applications, and substitute for fossil carbon carriers.^44^ In addition, biochar emerged as a low-cost, sustainable sorbent material with excellent adsorption properties; for instance, it can be used for the purification of flue gases through adsorption.^45^

The surface properties of biochar depend on employed raw materials and production conditions, in turn influencing the sorption capacity of the obtained solid.^46^ Biochar can be activated to increase its sorption capacity by introducing specific functional groups (functionalization) or inducing the growth of the specific surface area. Those activations can occur: (i) physically, for example, CO_2_ and steam are used as activation agents to control surface characteristics such as specific surface area, specific pore volume, and pore size distribution;^47,48^ (ii) chemically, by metal impregnation,^49,50^ heteroatom doping,^51^ and treatments with acidic/alkaline species such as HCl, H_2_SO_4_, H_3_PO_4_, KOH, and ZnCl_2_.^43,52–54^

In this framework, the present manuscript investigates PSA for CO_2_-capture on innovative biochar from vineyard pruning pellets, as obtained by the pyro-gasification at two Equivalence Ratios (*ER* = 0.15 or 0.30),^43^ and with additional chemical activation.^55^ Vineyard pruning waste was chosen because it is an interesting agro-industrial byproduct, available in large quantities in the Italian agro-industrial sector (e.g., in 2022 the theoretical potential availability of dry pruning from Italian vineyards was 1662 Gg).^43^

CO_2_-sorption capacity (*Y*) of the four biochar samples was measured in the tested pressure range of *P* = 5–9 bar_a_. Pressures were purposely low to investigate CO_2_ capture under conditions that would not require significant energy demand for gas repressurization. The *Y* responses were studied for preliminary explorative purposes by a 2^3^ factorial Design of Experiments (DoE)^56^ with *ER*, *P*, and “activation” as the investigated factors. The analysis of variance (ANOVA) was used to objectively determine the significance of main factor effects and interaction effects; an empirical mathematical regression model for the *Y* response surfaces was developed and validated. The reliability of that empirical model was evaluated against traditional Langmuir-type equations regressed by fitting experimental data.

## 2 Materials and Methods

### 2.1 As-Received Biochar Samples

As-received biochar had been previously produced by pyro-gasification at 650 °C of vineyard pruning pellets, by ENEA (Italian National Agency for New Technologies, Energy and Sustainable Economic Development) in Trisaia headquarters.^43^ Further information on ENEA pyro-gasification facility and biochar production is available in ref 57.

Two as-received biochar samples were studied in this work, produced at *ER* of 0.15 or 0.30 and called *BC0.15ar* and *BC0.30ar*, respectively.^43^ It is worth to stress that *ER* of 0.15 and 0.30 are plausible extreme values for the study of substoichiometric oxidative thermochemical conversions of carbonaceous compounds; for example, Basu^58^ reported that the carbon conversion efficiency in a gasification process is close to its maximum at *ER* = 0.26, so 0.30 can be considered a relatively high oxidative *ER* value for thermochemical conversions, whereas 0.15 is significantly less oxidant. This choice allowed the study of the experimental influences of *ER* on the behavior of biochar.

### 2.2 Biochar Activation

The method proposed and detailed by Gallucci et al.^55^ was used to activate biochar samples, as schematized in Figure S1 of the Supporting Information. *BC0.15ar* and *BC0.30ar* were both: (i) first thermally treated with KOH; (ii) then, attacked by an HCl aqueous solution; (iii) in the end, washed with distilled water until neutralization of liquid effluent, and dried. Activated samples are hereinafter called *BC0.15act* and *BC0.30act*, respectively derived by the activation of *BC0.15ar* and *BC0.30ar*.

KOH granules (Sigma-Aldrich) and a 37 wt % HCl solution (VWR Chemicals) were used as starting materials for the activation procedure.

### 2.3 Characterization of Sorbent Materials

Proximate analysis, ultimate analysis and heating values of *BC0.15ar* and *BC0.30ar* are available elsewhere^43^ and those results were roughly similar to those found in the literature concerning the same kind of biomass.^59^

N_2_ physisorption isotherms were recorded at the N_2_ atmospheric boiling point by a NOVA1200e Porosimeter (Quantachrome). The Brunauer–Emmett–Teller (BET) method was used to estimate the specific surface area (*S*_BET_), and the Barrett–Joyner–Halenda (BJH) method for the specific pore volume (*V*_BJH_, from the desorption isotherm). Before N_2_ physisorption, each sample was degassed for 3 h at 100 °C.^55^

Particle size distribution (PSD) of all samples was obtained through laser diffraction (Malvern Mastersizer 2000 analyzer).

Scanning electron microscopy (SEM) was used to observe morphological and elemental-topographical features, using a Philips XL30 CP microscope, at 20 kV, detecting high-resolution backscattered Electrons (BSE). Energy dispersive X-ray spectroscopy (EDS) was used to identify the local elemental composition of samples observed by SEM.

### 2.4 Experimental PSA Rig and Procedures

To evaluate the CO_2_-sorption capacity of samples, continuous dynamic PSA tests were performed in a bench-scale packed bed under industrially relevant conditions.

Figure 1 shows the schematization of the PSA experimental rig. The packed-bed reactor consisted of a 1/2 in. AISI 316L steel tube (ID = 0.91 cm, 55 cm long). Upstream, mass flow controllers (MFC, Figure 1) BRONKHORST regulated the N_2_ and CO_2_ inlet flow rates. Downstream, a BRONKHORST pressure controller (PC, Figure 1) determined the reactor set-point pressure (*P*) and a SIEMENS ULTRAMAT 23 gas analyzer measured the instantaneous outlet CO_2_ concentration (*c*_CO2,out_). A personal computer recorded *c*_CO2,out_ with a sampling period of 2 s. In order to ensure a proper minimum working flow rate to SIEMENS ULTRAMAT 23, a constant N_2_ flow rate (600 N mL/min) was fed at this analyzer inlet. N_2_ (internal standard) (grade 5.5) and CO_2_ (grade 4.0) bottles were used to carry out the PSA tests.

**Figure 1:**
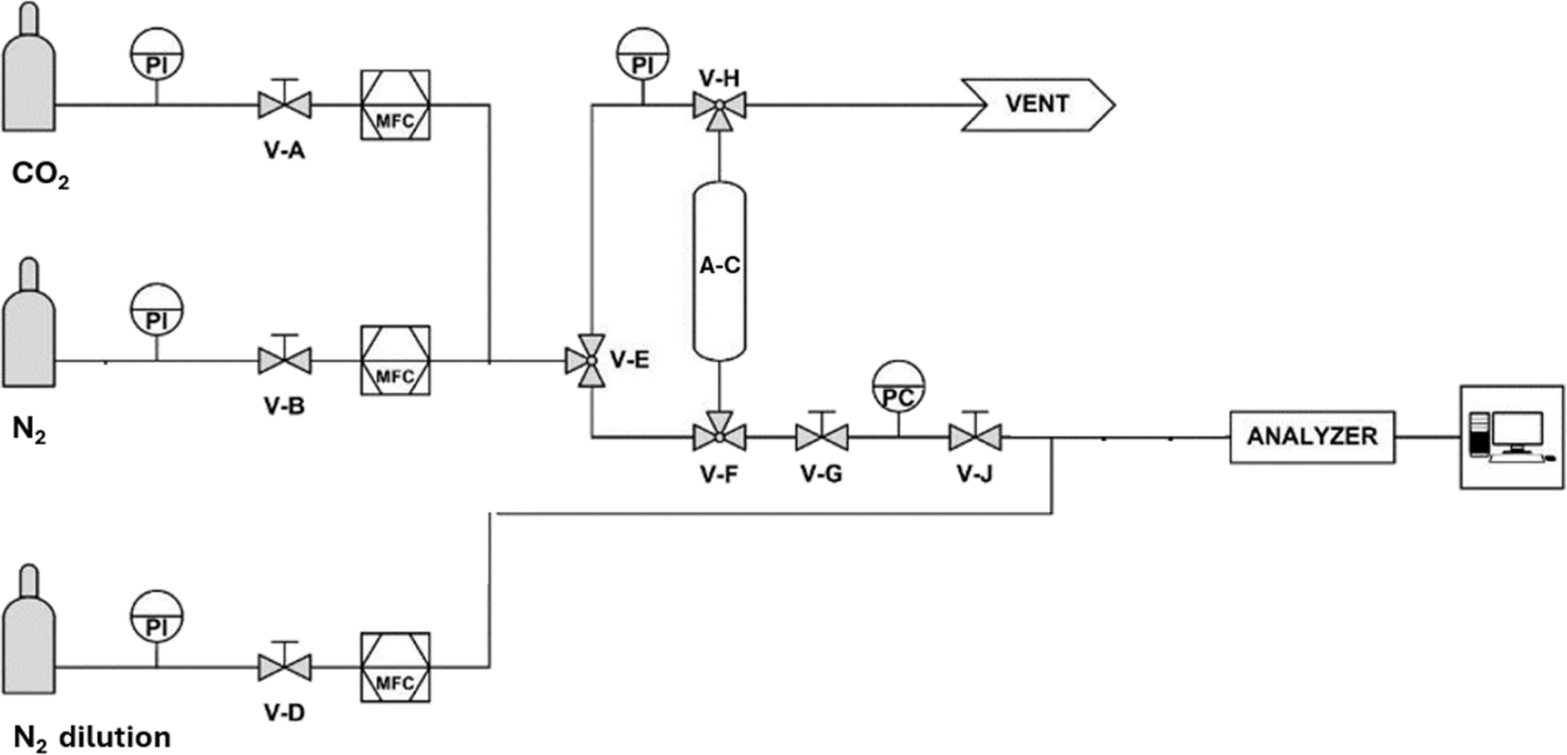
Schematization of the experimental laboratory-scale rig for PSA tests.

All the solid sorbent samples were sieved to obtain particles between 106 and 355 μm to decrease the influence of intraparticle mass transfer phenomena. For blank tests with an inert bed, glass beads with the same dimensional range were used.

PSA tests were performed at room temperature, at three different pressures (*P* = 5, 7, or 9 bar_a_), on an Active Column (A–C, Figure 1) containing a packed-bed of sieved biochar of about 0.8–1.0 g (i.e., about 2 cm^3^). The pressure drops in the A–C were preliminarily estimated using the Ergun equation^60^ and resulted negligible (on the order of 10^–2^ bar for a bed approximately 3 cm high). Furthermore, the adsorbent bed can be approximated as isothermal due to dimensions and test conditions. In the case of blank tests, the volume of biochar was substituted by an equivalent volume of the inert glass beads. During the adsorption phase of PSA tests, 184 N mL/min of gas with 46.5 vol % of CO_2_ (*c*_CO2,in_) in N_2_ (carrier gas)^55^ were fed downward to A–C at the chosen *P*. The CO_2_ adsorption phase at *P* was carried out until a stable plateau occurred with *c*_CO2,out_ equaling *c*_CO2,in_. The regeneration phase of the A-C was then carried out by an instantaneous pressure swing from *P* down to atmospheric pressure while 250 N mL/min of N_2_ was flowing upward. The management of valves (V– in Figure 1) to implement PSA cycles was manual, as detailed in the Supporting Information. Five PSA cycle replications at each *P* (5, 7, and 9 bar_a_) were performed for all biochar samples and the blank test.

### 2.5 Data Elaboration

#### 2.5.1 Quantification of *Y*_exp_

Experimental values of CO_2_-sorption capacity of biochar samples (*Y*_exp_) were quantified by mole balances, calculating the amount of adsorbed CO_2_ in each adsorption phase of PSA cycles. Outlet CO_2_ molar flow rates for mass balances were calculated by assuming that the overall N_2_ flow rate was the internal inert standard. Details of this quantification method are described elsewhere,^61^ based on First Order With Dead Time Model (FOWDTM) for gas mixing.^62^ Blank tests at each *P* allow quantifying the moles holdup of CO_2_ in the experimental system without adsorption, by means of characteristic *c*_CO2,out_ curves as a function of time, and therefore by the corresponding curves of CO_2_ outlet flow rate. The same kind of curve from the adsorption phase of PSA tests at *P* allows quantifying the mole holdup of CO_2_ in the experimental system that contains a biochar sample. The FOWDTM considers the delay between the *c*_CO2,out_ adsorption curves and the blank as proportional to the CO_2_ captured by the sorbent material in the column.^62^ Therefore, *Y*_exp_ is the difference between the two CO_2_ mole holdups of blank and test with a sorbent, per mass unit of sorbent.

#### 23 Factorial DoE and the Regression Model

Considering that no decreasing performances were noted in cyclic PSA tests, a 2^3^ factorial DoE^56^ was used to frame the measured responses of *Y*_exp_, and study them for explorative purposes. *P*, *ER,* and “activation” were selected as the three design factors, respectively, named A, B, and C in the statistical analysis of *Y*_exp_ data. The chosen levels were 5 bar_a_ or 9 bar_a_, 0.15 or 0.30, “as-received” (no activation) vs “activated” (Table 1).

**Table 1:** Factor and Levels Investigated in the 2^3^ Factorial DoE Carried Out on PSA CO_2_-capture Tests

effect	quantity	low level	high level
A	*P* (bar_a_)	5	9
B	*ER* (−)	0.15	0.30
C	activation	no (“*ar*”)	yes (“*act*”)

The significance of factors’ main effects and interactions was evaluated by ANOVA for each biochar sample (*F*-tests with α = 5% significance level). The empirical “linear model with main-effects and interactions” (eq 1) was assumed for the regression of the CO_2_-sorption capacity responses, with *Y*′ as the empirically modeled response of CO_2_-sorption capacity: values of β_*i*_ coefficients in eq 1 were assumed excluding main effects and interactions that were nonsignificant according to ANOVA. The independent quantitative variables *X*_*A*_ and *X*_*B*_ (eq 1), corresponding to the factors A (*P*) and B (*ER*), were expressed in encoded form (−1; +1) by the normalizations in eq 2. As for factor C (“activation”), the independent variable *X*_*C*_ is categorical, encoded as “–1” if biochar is “as-received” (i.e., not activated) and “+1” if biochar is “activated”. Response surfaces for *Y*′ in the form of eq 1 were obtained and analyzed.

$$Y^{\prime }=\beta_{0}+\beta_{A}X_{A}+\beta_{B}X_{B}+\beta_{C}X_{C}+\beta_{AB}X_{A}X_{B}+\beta_{AC}X_{A}X_{C}+\beta_{BC}X_{B}X_{C}+\beta_{ABC}X_{A}X_{B}X_{C}$$ 1

$$X_{A}=\frac{P-7}{2};\;X_{B}=\frac{ER-0.225}{0.075}$$ 2

The empirical linear regression model with interactions was validated by comparisons with the experimental data at 7 bar_a_, that were not used in the regression: the parameter δ (eq 3) was an index of the deviation between *Y*_exp_ and *Y*′ at the validation value of 7 bar_a_.

$$\delta ={\frac{Y^{\prime }-Y_{\exp}}{Y_{\exp}}|}_{7{\mathrm{bar}}_{a}}$$ 3

#### 2.5.3 Study of *Y* by the Langmuir Isotherm

A behavior described by a Langmuir-type isotherm (eq 4) was assumed as a mechanistic equilibrium model of *Y*. In order to distinguish between the different modeling approaches, values of *Y* calculated by the Langmuir isotherm were symbolized by *Y*″.

$$Y^{\prime \prime }=\frac{Y_{\max}\cdot p_{{\mathrm{CO}}_{2}}}{K+p_{{\mathrm{CO}}_{2}}}$$ 4

The Lineweaver–Burk (double reciprocal) linearization method^63^ was used to regress for each material the Langmuir parameters *Y*_MAX_ and *K*, fitting the *Y*_exp_ measurements as functions of CO_2_ partial pressure (*p*_CO2_ = 0.465*P*).

To compare the CO_2_-sorption capacities obtained by the Langmuir model (*Y*″, eq 4) and empirical linear model (*Y*′, eq 1), the deviation parameter φ (eq 5) was introduced. This parameter may serve to evaluate the extrapolation potential of the empirical model in eq 1.

$$\varphi =\frac{Y^{\prime }-Y^{\prime \prime }}{Y^{\prime \prime }}$$ 5

## 3 Results and Discussion

### 3.1 Characterization of Solid Samples

Table 2 shows the results of the BET-BJH analyses. The *S*_BET_ of as-received materials (*BC0.15ar* and *BC0.30ar*) was negligible with respect to the detection limits of the method (Table 2). On the other hand, activated materials (*BC0.15act* and *BC0.30act*) experienced a significant increase in *S*_BET_, reaching the order of magnitude of 10^2^ m^2^ g^–1^ (Table 2): the activated material with the highest *ER* showed the highest *S*_BET_ (*BC0.30act*, 756 m^2^ g^–1^ in Table 2). As for the pore volume, *V*_BJH_ significantly increased after the activation procedure (Table 2, *BC0.30act* vs *BC0.30ar, BC0.15act* vs *BC0.15ar*).

**Table 2:** Experimental Results of N_2_ Physisorption Isothermal Measurements: BET Specific Surface Area (*S*_BET_) and BJH Cumulative Desorption Pores Volume (*V*_BJH_)

material		BC0.15ar	BC0.30ar	BC0.15act	BC0.30act
*S*_BET_	(m^2^ g^–1^)	1	0	608	756
*V*_BJH_	(cm^3^ g^–1^)	0.011	0.005	0.056	0.043

Figure 2 represents the particle size distribution (PSD) of the analyzed samples: compared to other samples, *BC0.15act* showed a peculiar PSD shifted toward smaller diameters and with a more important fraction of finer particles (Figure 2), even after preparatory manual sieving between 106 and 355 μm. This suggested an intrinsically higher fragility.

**Figure 2:**
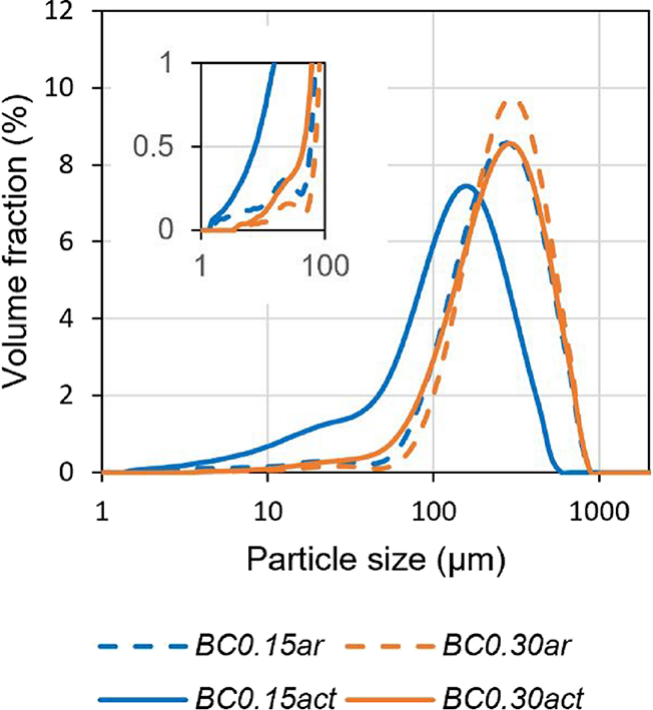
Particle size distributions of biochar samples.

Figure 3 shows the SEM micrographs of biochar samples: the morphology of as-received (*BC0.15ar* and *BC0.30ar*) and activated samples (*BC0.15act* and *BC0.30act*) confirms the trend indicated by BET-BJH results (Table 2), i.e., the activation treatment (Section 2.2) induces an increase of biochar porosity. From the elemental point of view, SEM-EDS maps (Figure 4) highlighted that the activation procedure had a washing action (*BC0.15ar* Figure 4a vs *BC0.15act* Figure 4b; *BC0.30ar* Figure 4c vs *BC0.30act* Figure 4d). In addition, the biochar with *ER* of 0.15 appeared to keep more mineral residuals after the activation (Figure 4b vs d and Figure 4e vs f), in which a more conspicuous presence of Mg, Ca, and K emerged. Further details about SEM-EDS are in the Supporting Information.

**Figure 3:**
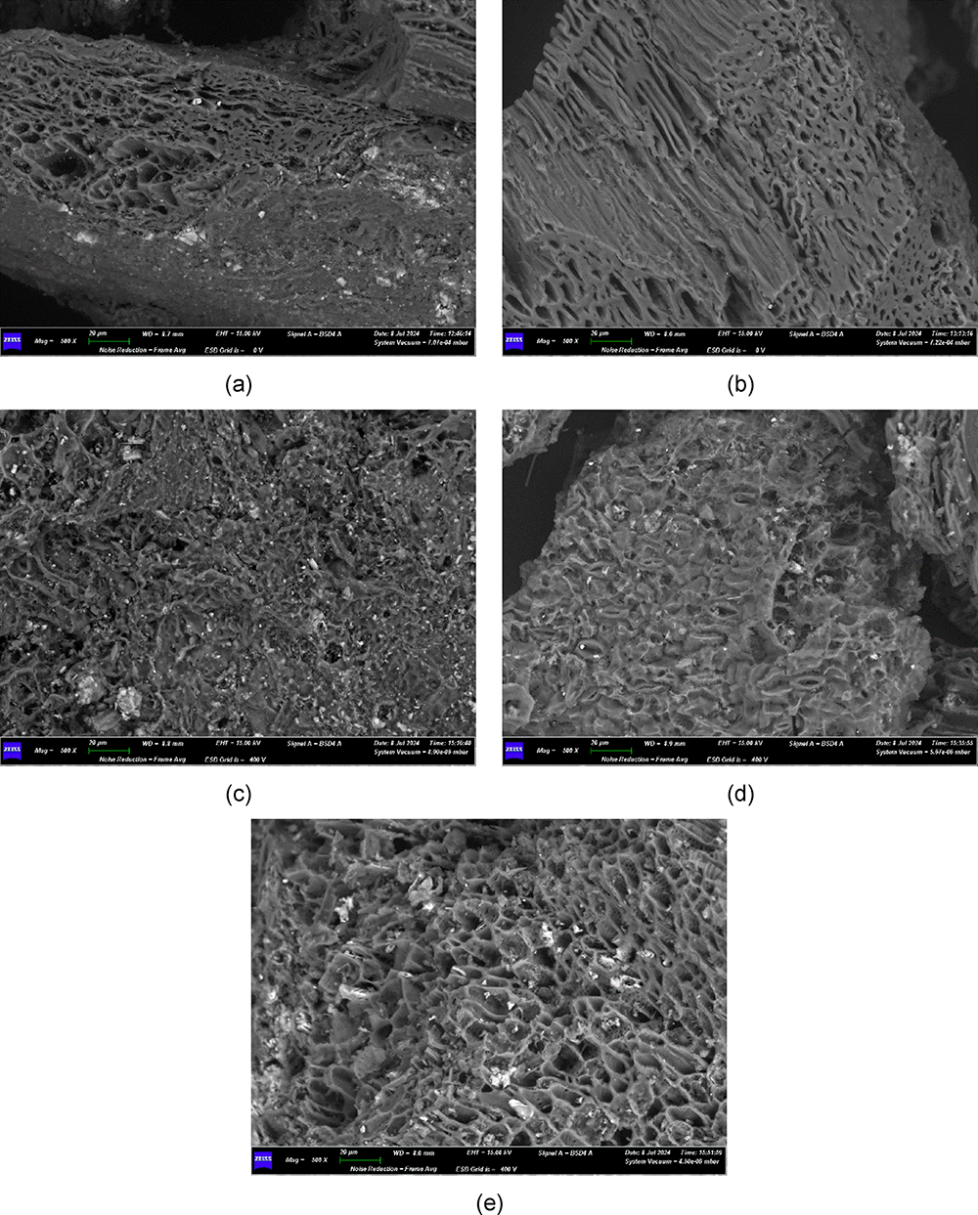
SEM micrographs of synthesized biochar samples: (a) *BC0.30ar*; (b) *BC0.30act*; (c) *BC0.15ar*; and (d) *BC0.15act*. SEM micrograph post-test: (e) *BC0.15act*. Magnification of all micrographs at 500×.

**Figure 4:**
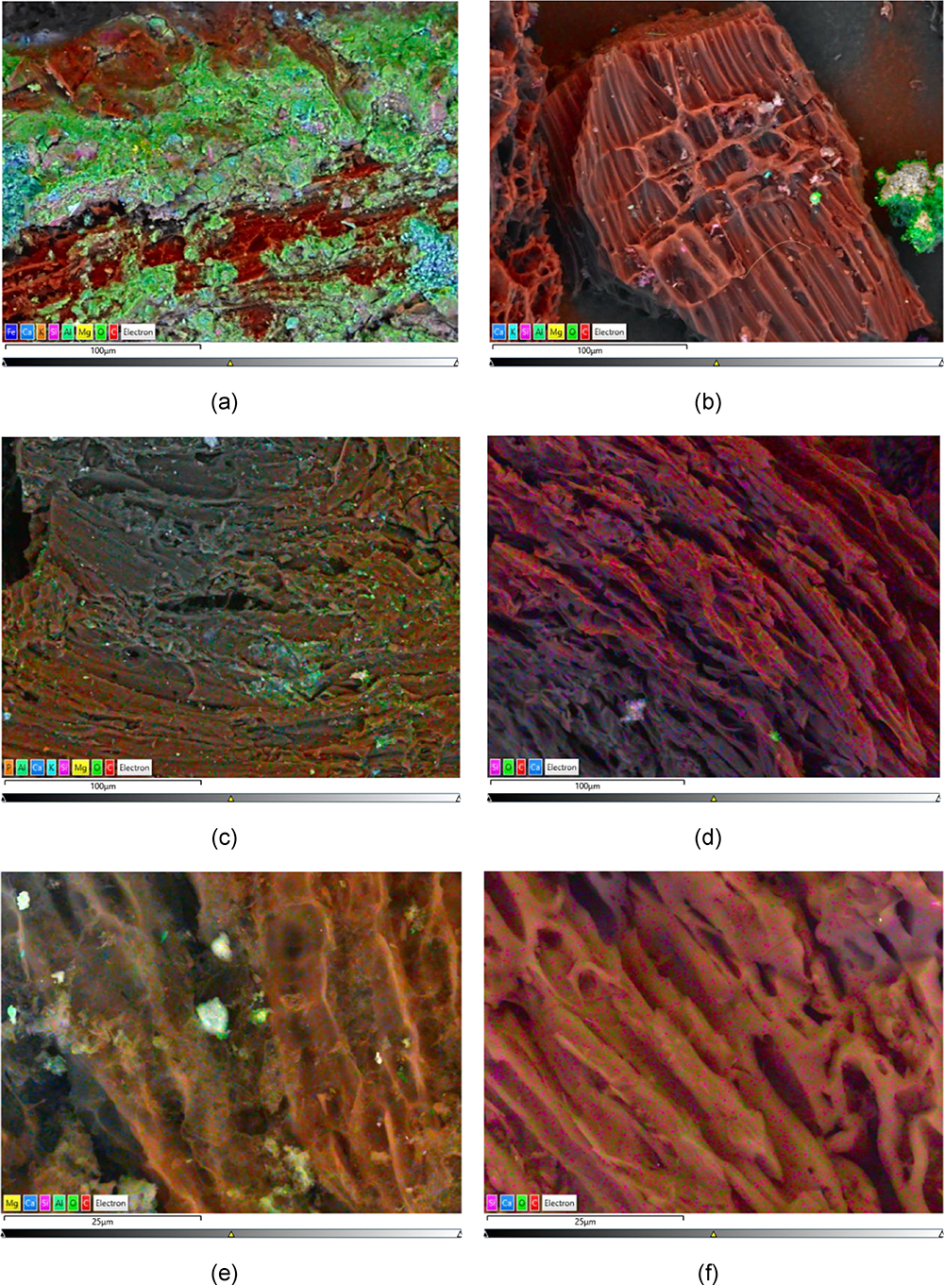
SEM-EDS maps of biochar samples: (a) *BC0.15ar* (Scale bar: 100 μm, Magnification: 500×); (b) *BC0.15act* (Scale bar: 100 μm, Magnification: 500×); (c) *BC0.30ar* (Scale bar: 100 μm, Magnification: 500×); (d) *BC0.30act* (Scale bar: 100 μm, Magnification: 500×); (e) *BC0.15act* (Scale bar: 25 μm, Magnification: 2000×); (f) *BC0.30act* (Scale bar: 25 μm, Magnification: 2000×) Single-map of elements are given in the Supporting Information.

### 3.2 CO_2_ Adsorption in PSA Tests

Figure S3 in the Supporting Information shows an example of the general features of all outlet CO_2_ flow rate curves recorded during PSA replications at the three *P* levels, compared with respective blanks: (i) the curves always had sigmoidal shape, in agreement with the literature^43,55^ ; (ii) the higher the *P*, the greater the delay in the release of CO_2_ as also observed elsewhere^40,62^ ; (iii) the response curves of the five PSA cycle replications (PSA*n*, with *n* = 1,...,5 in Figure S3) were almost perfectly overlapped without time trends, that is, the sample was always equally regenerated in the five cycles exerted at each *P*, so they can be considered five replications of the same phenomenon and treated by the DoE described in Section 2.5.2. This behavior indeed suggests that no irreversible chemisorption (or any other detrimental/incremental phenomenon) occurred at room temperatures and operating pressures. The observed adsorption phenomenon is purely physical; the regeneration procedure with N_2_ at atmospheric pressure was sufficient to completely restore the properties of the investigated sorbent materials five times. However, some degree of material degradation was observed in the literature during PSA cycles. Ferella et al.^40^ observed that the highest adsorption capacity of zeolites was achieved in the first PSA cycle, with a decrease in subsequent cycles (although the decrease was only 13%). This trend is also observed by Kacem et al.^64^ about zeolites and attributed to mechanical wear of its structure, the accumulation of impurities, and potential oxidation reactions; in the same study,^64^ activated carbons showed complete reversibility of PSA cycles by simple pressure modulation. In the case of carbon molecular sieves, industrial application is ensured in the literature for the entire lifetime of a PSA plant.^65^ Based on the observations, the materials studied in this work ensure durability comparable to that of activated carbon and better than some zeolites. However, the studies conducted at this scale cannot exclude the possibility of performance deterioration or loss of mechanical stability of the materials in long-term industrial applications. It should still be noted that an appropriate design of the fixed-bed PSA column can help to minimize the mechanical degradation of the material. Moreover, operating at low pressures can reduce the mechanical stress. Figure 5 shows *Y*_exp_ data as functions of exerted *P*. The values of represented data are summarized in Table S1 of the Supporting Information. For all samples, *Y*_exp_ increased along with *P* as also explained by Kacem et al.^64^

**Figure 5:**
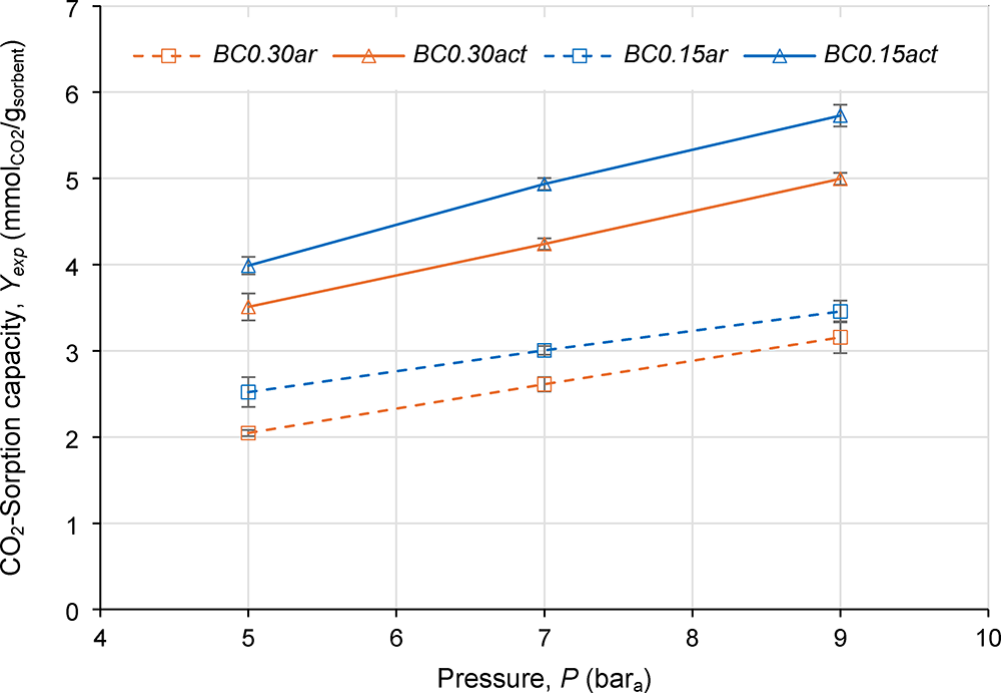
Experimental CO_2_-sorption capacities (*Y*_exp_) as functions of pressure (*P*); error bars = 95% confidence intervals.

The as-received biochar sorbents (*BC0.15ar* and *BC0.30ar*) showed a close *Y*_exp_ (Figure 5). Activated biochar samples, *BC0.15act* and *BC0.30act*, exhibited the highest *Y*_exp_ compared to as-received samples (Figure 5). These results can be attributed to the significant increase in *S*_BET_ obtained after activation (Table 2). However, *S*_BET_ cannot be the only factor that determined *Y*_exp_ variations among samples: even though *BC0.15act* had a lower *S*_BET_ than *BC0.30act* (Table 2), the *Y*_exp_ of *BC0.15act* was higher than the *Y*_exp_ of *BC0.30ar* (Figure 5). The higher *Y*_exp_ obtained in *BC0.15act* compared to *BC0.30act* can be attributed also to small quantities of spurious elements in the sorbent material, such as Mg, K, Ca, P, and S (shown in SEM-EDS of Figure 4d,e), that can act as additional active sites for the adsorption of gas molecules, as mentioned by Park et al.^66^ Improving effects of spurious elements on CO_2_ uptake is described in the literature also for noncarbonaceous sorbent materials.^67^ Some of those spurious elements were found more significantly in *BC0.15act* than in *BC0.30act* by the SEM-EDS maps (Figure 4), due to the more important amounts of mineral matter in *BC0.15ar* (Figure 4a). Therefore, the simultaneous significant increase of *S*_BET_ due to activation (Table 2) and the more abundant presence of spurious elements (Figure 4e) can explain the fact that *BC0.15act* exhibited the highest *Y*_exp_ among the tested materials. As further evidence in this sense, all the biochar samples with *ER* = 0.15 showed higher *Y*_exp_ than the respective ones with *ER* = 0.30 (Figure 5).

*Y*_exp_ of tested biochar samples was comparable to or even higher than that of commercial sorbent materials studied by Ferella et al.^40^ and Gallucci et al.,^55^ such as activated carbon, silica gel and zeolite 13X, which respectively have CO_2_-sorption capacities of 1.97, 0.64, and 0.65 mmol_CO2_/g_sorbent_ at 5 bar_a_. Results were also comparable with measurements of Georgieva et al.^67^ on merlinoite zeolites in Na and K forms that show CO_2_ uptakes at 5 bar and 298 K of 4.8 mmol_CO2_/g_sorbent_ and 4.2 mmol_CO2_/g_sorbent_, respectively.

*BC0.15act* was selected for further SEM characterization after tests because it gave the best CO2-sorption performance. No morphological differences were found between the *BC0.15act* sample before PSA (Figure 3d) and *BC0.15act* after PSA (Figure 3e); this is a further observation suggesting the potential good morphological stability of the material.

Overall, the reuse of this vineyard pruning waste as sorbent materials for PSA for CO_2_-capture appeared to be feasible.

### 3.3 Statistical Analysis and Modeling

The statistical analysis of *Y*_exp_ data was performed according to the 2^3^ factorial DoE described in Section 2.5.2, with the treatments identified in Table 3 by the standard notation (e.g., “(−1)” = treatment with factors A, B, and C at their lower levels; “a” = treatment with factor A at its higher levels and factors B and C at their lower; “abc” = treatment with factors A, B, and C at their higher levels).

**Table 3:** Treatments of the 2^3^ Factorial DoE with Five CO_2_ Capture Replications (PSA*n*, with *n* as the Number of Replication)

treatment	*P*(bar_a_)	*ER*	activation	*Y*_exp_(mmol_CO2_/g_sorbent_)				
				PSA1	PSA2	PSA3	PSA4	PSA5
(−1)	5	0.15	no	2.63	2.47	2.57	2.60	2.33
a	9	0.15	no	3.42	3.38	3.60	3.40	3.49
b	5	0.30	no	2.05	2.06	2.01	2.07	2.04
ab	9	0.30	no	3.25	3.17	3.22	3.22	2.92
c	5	0.15	yes	3.99	4.06	3.89	4.06	3.95
ac	9	0.15	yes	5.71	5.88	5.70	5.65	5.70
bc	5	0.30	yes	3.49	3.54	3.48	3.36	3.67
abc	9	0.30	yes	5.03	5.00	4.92	5.05	4.98

Considering the full reversibility of PSA cycles under all tested conditions, the five cycles at each treatment were considered here as statistical replications (Table 3). The consequent ANOVA on *Y*_exp_ is summarized in Table 4. The highest *Y*_exp_ were obtained in correspondence with the “ac” and “abc” treatments, that is, *P* = 9 bar_a_, with both *ER* values and only for the activated materials.

**Table 4:** ANOVA (Significance Level α = 5%) Connected to the 2^3^ Factorial DoE with Five CO_2_ Capture Replications (Variance of Experimental Error Calculated by the Five Replications)

effect	effect value	sum square	degrees of freedom	mean square	*F* value	1–*P*-value	significant
A	1.32	17.36	1	17.36	1973.83	1.0000	yes
B	–0.50	2.47	1	2.47	280.46	1.0000	yes
AB	–0.02	0.004	1	0.004	0.46	0.4662	no
C	1.76	31.00	1	31.00	3523.40	1.0000	yes
AC	0.29	0.87	1	0.87	98.90	0.9999	yes
BC	–0.11	0.12	1	0.12	13.60	0.9992	yes
ABC	–0.11	0.12	1	0.12	13.06	0.9990	yes
error		0.2815	32	0.0088			

The results of the ANOVA showed that the main effects of A, B, C and the interactions AC, BC, and ABC were significant, whereas AB was not significant, all at α = 5% significance level (Table 4). By observing the related Pareto plot (Figure 6), it is possible to note that

1) Main effects were all significant: *P* (factor A) and “activation” (factor C) had the by-far two largest effects on the response *Y*, both positive, whereas the effect of *ER* (factor B) was negative with lower magnitude; this was in agreement with the experimental fact that in all cases the “activation” and increase of *P* gave higher *Y*_exp_, whereas the increase in *ER* led to a slight decrease in *Y*_exp_ (Figure 6).
2) As for interactions, the one between *P* and “activation” (AC) was the most significant, with a low positive effect, i.e., the effect of pressure was slightly amplified with the activation of the material, as shown by the interaction plot in Figure 7a: the effect of *P* (factor A) on *Y* was more important when the biochar was activated (C+, *act*), compared to one for the biochar as-received (C–, *ar*).
3) The interaction between *ER* and “activation” (BC) was negative with magnitude lower than AC as also evidenced by the interaction plot in Figure 7b: for the activated biochar (C+, *act*) the increase in *ER* (factor B) led to a decrease of CO_2_-sorption capacity slightly more pronounced than for as-received biochar (C–, *ar*).
4) The ternary interaction ABC was negative and with a magnitude similar to BC.

**Figure 6:**
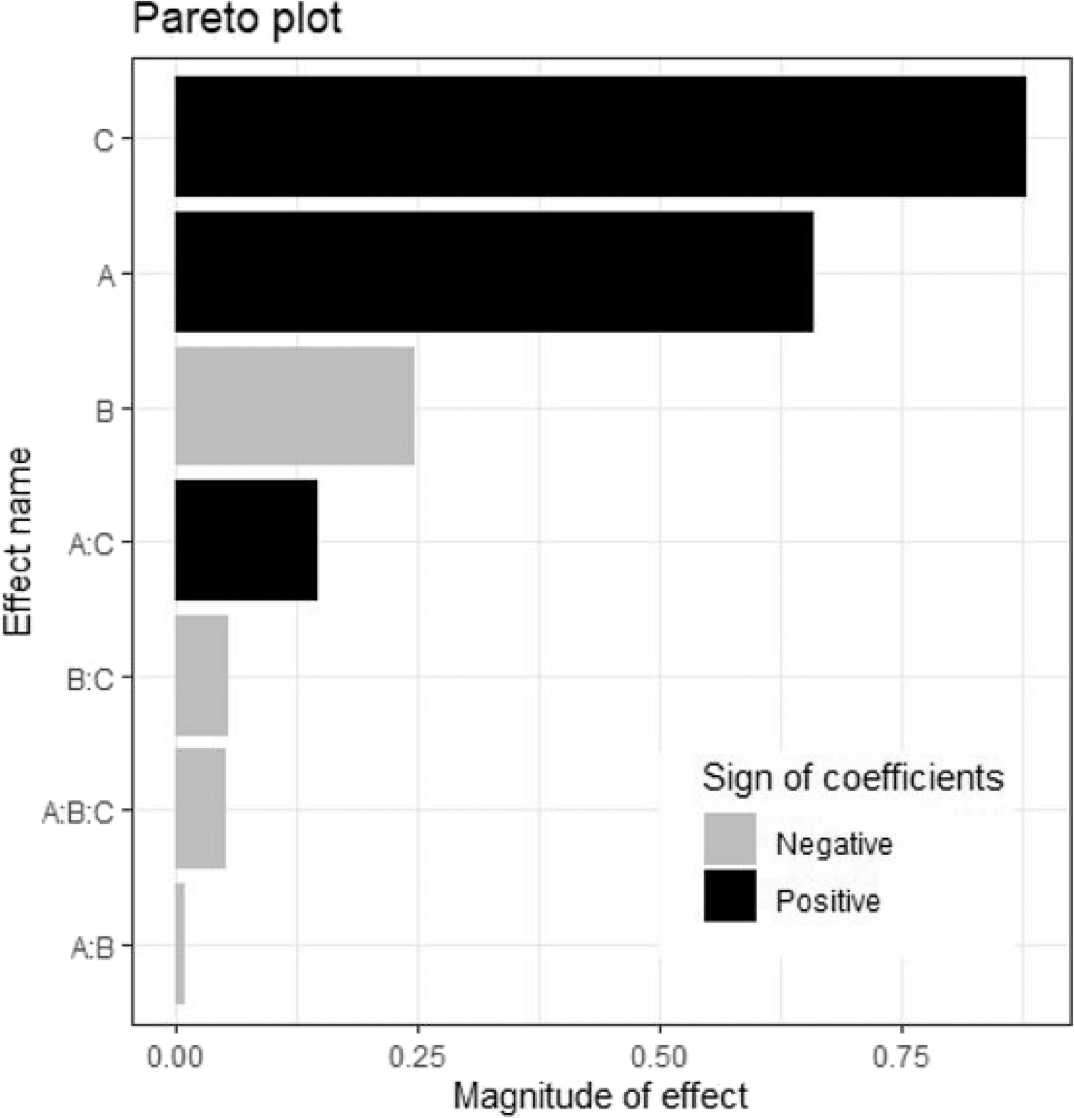
Pareto plot to evaluate the magnitude of effects (main effects and interaction), with factors A = *P*, B = *ER*, and C = “activation”.

**Figure 7:**
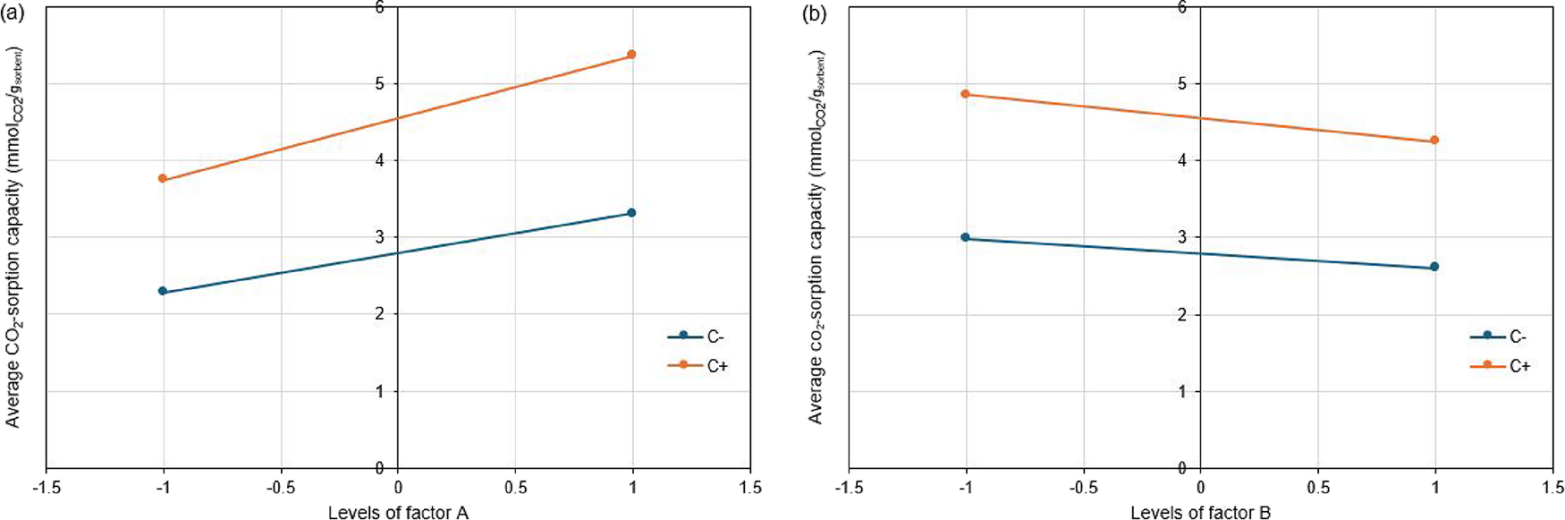
Interaction plots: interaction AC (a), and interaction BC (b), with factors A = *P*, B = *ER*, C = “activation”, C– = C at its lower level (*ar*, as-received), and C+ = C at its higher level (*act*, activated).

#### 3.3.1 Surface Responses and Validation of Their Empirical Regression Model

Based on the *Y*_exp_ results, framed in the 2^3^ factorial DoE and the related ANOVA, the β_*i*_ parameters in eq 1 were regressed excluding the nonsignificant effect β_AB_ (Table 4). Eq 6 shows the corresponding empirical linear model with interactions for *Y*′ (expressed in mmol_CO2_/g_sorbent_). Note that the parameter values (β_A_, β_B_, β_C_, β_AC_, β_BC_ and β_ABC_) are half of the effect values found by ANOVA (Table 4).^68^

$$Y^{\prime }=3.68+0.66X_{A}-0.25X_{B}+0.88X_{C}+0.15X_{A}X_{C}-0.055X_{B}X_{C}-0.054X_{A}X_{B}X_{C}$$ 6

The *Y*_exp_ values were represented in a scatterplot compared with the values of *Y*′ at the same conditions (Figure 8a). The empirical regression model (eq 6) was suitable to explain the capture of CO_2_ by the studied biochar samples as a function of *P*, *ER* and biochar activation: the determination coefficient (*R* ^2^) of Figure 8a was 0.995, indicating that only 0.5% of the total variation around the average is not explained by the regressed eq 6.

**Figure 8:**
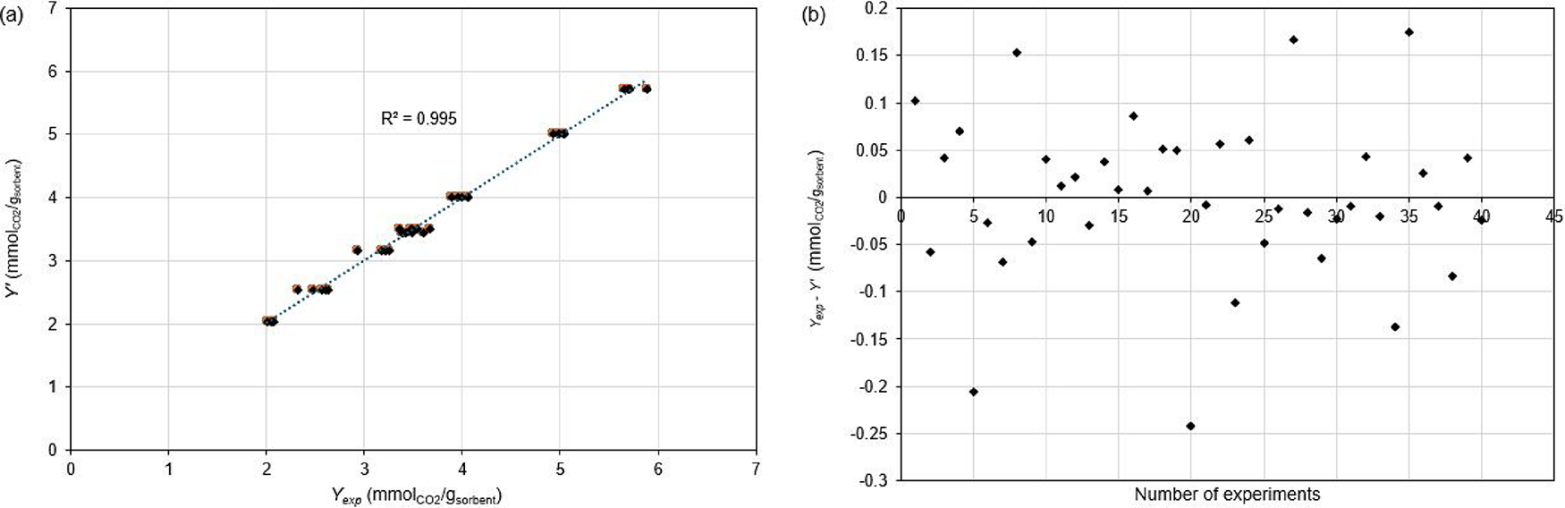
(a) Scatterplot of calculated CO_2_-sorption capacity (*Y*′) vs experimental CO_2_-sorption capacity (*Y*_exp_) (mmol_CO2_/g_sorbent_). (b) Residual plot (*Y*_exp_ – *Y*′) vs number of the experiments.

The analysis of residual values (*Y*′ – *Y*_exp_) was represented in the plot of Figure 8b: there were not evident trends or patterns in residuals, confirming the suitability of the empirical linear regression model in eq 6.

To validate the empirical regression model (eq 6), the deviation parameter δ was calculated (eq 3); δ compares the experimental data obtained at the intermediate *P* of 7 bar_a_ (not used in the regression) with the related *Y*′ responses calculated by the regression model (eq 6 with *X*_A_ = 0). The empirical linear regression model (eq 6) predicted *Y*′ at 7 bar_a_ within δ values of +2 and −3%, as also shown in Figure S4 of Supporting Information.

Figure 9 shows the response surfaces and contour plots of *Y*′ (eq 6) as functions of the effects A (*P*) and B (*ER*), for the biochar samples in as-received (Figure 9a,b) and activated (Figure 9c,d) states. In both cases, the direction to increase the CO_2_-sorption capacity involves the increase of *P* and the decrease of *ER* (Figure 9). The calculated *Y*′ values of as-received biochar samples were between 3.2 and 3.4 mmol_CO2_/g_sorbent_ at 9 bar_a_ and *ER* equaling 0.15, and dropped below 2.2 mmol_CO2_/g_sorbent_ at 5 bar_a_ and *ER* of 0.30 (Figure 9a,b). The *Y*′ values of activated biochar samples were higher (between 5.4 and 5.6 mmol_CO2_/g_sorbent_) at 9 bar_a_ and *ER* of 0.15, dropping below 3.6 and 3.8 mmol_CO2_/g_sorbent_ at 5 bar_a_ and *ER* of 0.30.

**Figure 9:**
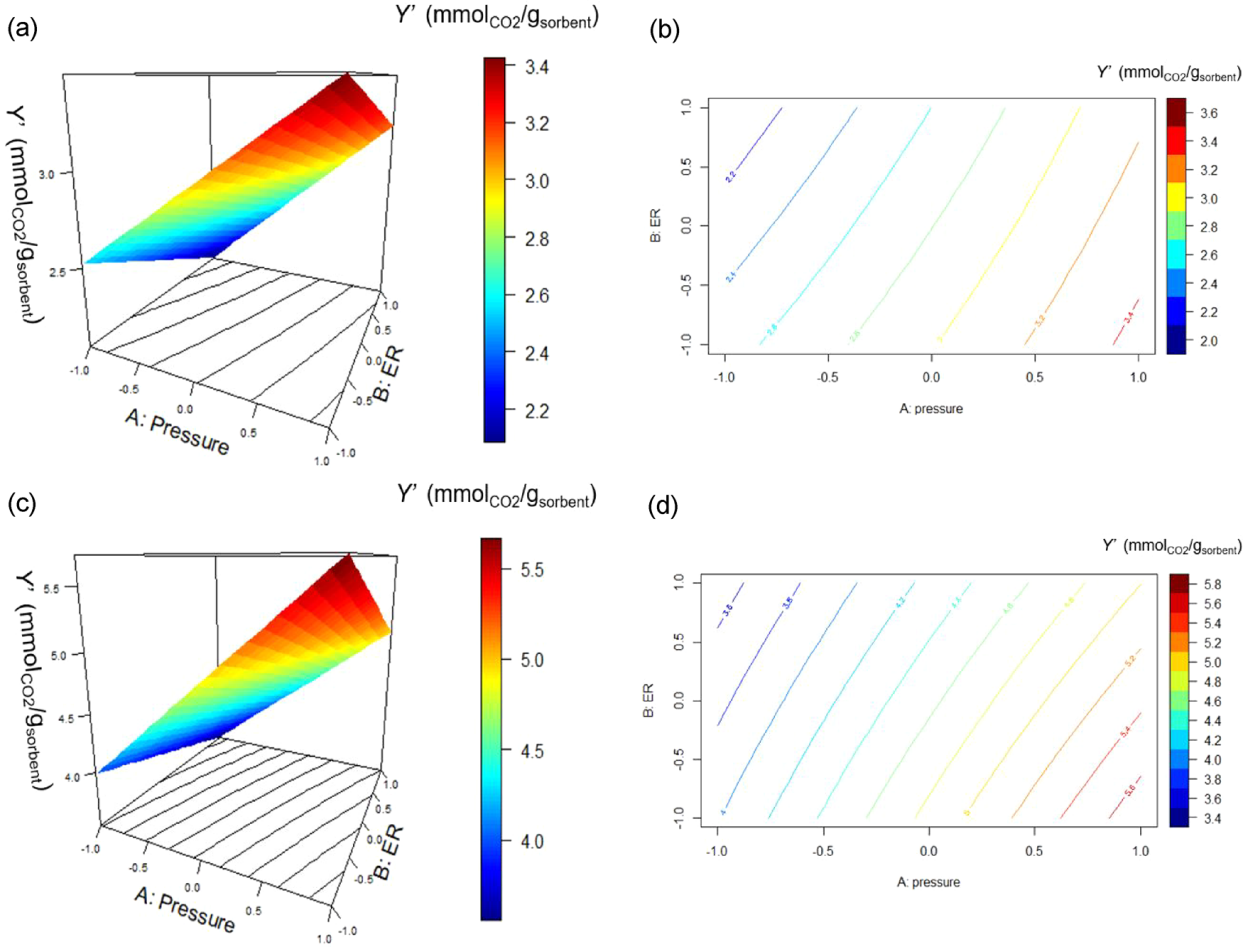
Regressed CO_2_-sorption capacity (*Y*′ (mmol_CO2_/g_sorbent_), eq 6) as a function *P* and *ER*: (a) response surface of as-received biochar, (b) contour plot of as-received biochar; (c) response surface of activated biochar, and (d) contour plot of activated biochar.

From the analysis of these results, one should be oriented to choose activated biochar produced at a low *ER* (0.15) and work at high pressure (9 bar_a_) to have the highest CO_2_-sorption capacity of the investigated system. This is in line with observations proposed in the first qualitative analysis of experimental results in Section 3.2.

The empirical model for evaluating *Y*′ serves as a simple and effective tool for determining the maximum amount of CO_2_ that can be captured by the investigated sorbent materials within a pressure range of 5–9 bar_a_ (a range compatible with industrial applications).^30–32^ Furthermore, the CO_2_ sorption capacity is crucial for the design of adsorption columns, as the amount of material required (which determines the column size and associated costs) directly depends on the quantity of CO_2_ captured by the chosen sorbent material.^69^ In an industrial scale, pellets could be used instead of the granular solid investigated in this work. However, this would have a greater influence on the capture kinetics than on the maximum sorption capacity quantified by the empirical model.

#### 3.3.2 Study of Empirical Model Extrapolation by Comparison with Langmuir Regressed Isotherms

Parameters *Y*_MAX_ and *K* of the Langmuir isotherm (eq 4) were regressed for as-received and activated biochar samples, fitting the average CO_2_-sorption capacities obtained from PSA cycles at each pressure level. Results are given in Table 5.

**Table 5:** Regressed Parameters of the Langmuir Isotherm (Eq 4) Obtained from *Y*_exp_

	*Y*_MAX_(mmol_CO2_/g_sorbent_)	*K*(bar_a_)	*R* ^2^
*BC0.15ar*	6.897	0.251	0.9972
BC0.15act	13.175	0.188	0.9998
*BC0.30ar*	9.524	0.117	0.9991
*BC0.30act*	11.036	0.202	0.9947

Figure 10 compares CO_2_-sorption capacities of the as-received and activated biochar samples, calculated by the Langmuir isotherm curves (*Y*″*,* eq 4 with *Y*_MAX_ and *K* in Table 5) and the corresponding empirical linear regression straight lines (*Y*′, eq 6). Both the Langmuir isotherm and the linear regression model fit well the experimental data within the investigated pressure range (Figure 10).

**Figure 10:**
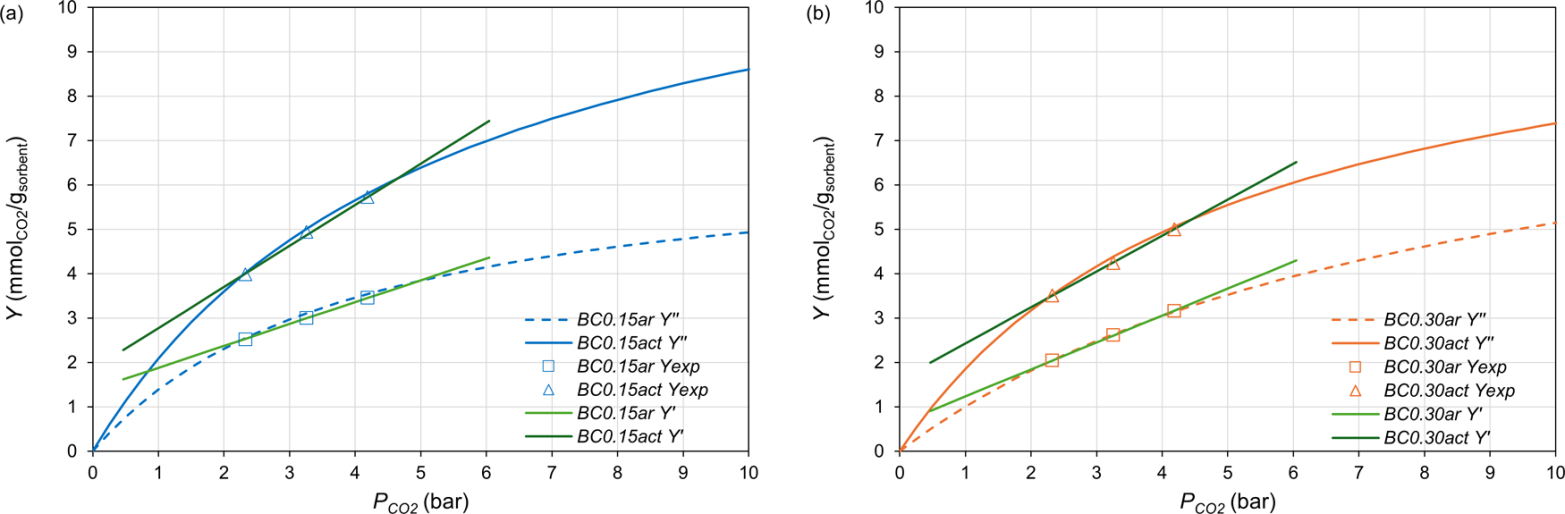
CO_2_-sorption capacity (*Y*) vs *p*_CO_2__ predicted by empirical linear regression model (eq 6) and Langmuir isotherm model (eq 4), for: (a) *BC0.15ar* and *BC0.15act*; (b) *BC0.30ar* and *BC0.30act*.

The comparisons in Figure 10 validate an extrapolation potential of the empirical linear regression model (*Y*′, eq 6). Under the assumption that the acceptable threshold of deviation φ (eq 5) between *Y*′ and *Y*″ is ± 5%, the extrapolation of the linear regression model (eq 6) produced acceptable *Y*′ predictions in the extended *P* range from 4 to 11 bar_a_ (to *p*_CO2_ = 1.86 bar and up to *p*_CO2_ = 5.12 bar), as also shown in Figure S5 of the Supporting Information.

The proposed empirical linear regression model (eq 6) resulted as a simple and predictive tool for calculating from 4 to 11 bar_a_ the CO_2_-sorption capacity of the biochar samples *BC0.15ar*, *BC0.15act*, *BC0.30ar*, and *BC0.30act*.

### 3.4 Final Remarks

This study must be intended as a preliminary laboratory-scale investigation that proved the potential of biochar and related activation procedures to develop sustainable forms of CO_2_ capture.

The physical nature of the samples investigated is an aspect worthy of being commented on. The shape in sieved powder is legitimated by the experimental laboratory-scale adopted in this work and the aim of limiting transport phenomena influences to make all materials work at their best (i.e., saturation); however, it is sensible to suppose that future mockup or pilot-scale studies should assume that the sorbent material that constitutes the active beds of PSA vessels should be industrially formed (e.g., shaped in pellets). Therefore, on the other hand, this does not influence the validity of the proposed modeling because it concerns saturation of sorbents (therefore an equilibrium threshold representative of the material).

The comparison between the empirical regression model of surface response and the mechanistic model of Langmuir adsorption equilibrium should be taken as a remark against the uncontrolled extrapolation of simplified empirical linear models applied to phenomena intrinsically nonlinear.

## 4 Conclusions

Biochar samples, previously obtained from pyro-gasification of agro-industrial waste (vineyard pruning) at two equivalence ratios (0.15 and 0.30), were chemically activated by a treatment with KOH/HCl.

As-received and activated biochar samples were tested for PSA applied to capture CO_2_ at 5, 7, and 9 bar_a_. All biochar samples had CO_2_-sorption capacities comparable or even higher than those reported in the literature. Activated materials had by far the highest surface areas; this led them to have the highest CO_2_-sorption capacities at all tested pressures with supposed minor influences from spurious mineral elements in traces.

The framing of CO_2_-capture capacities in a 2^3^ factorial design of experiments determined that activation and then pressure had the greatest and positive main effects on biochar CO_2_-sorption capacity; only the interaction between the pressure and equivalence ratio was not significant. The obtained empirical linear regression model of CO_2_-sorption capacity (first-order main effects with interactions) can be extrapolated in the range of 4–11 bar_a_ with deviations lower than ±5% from Langmuir isotherm behavior for both as-received and activated biochar samples.

Therefore, predictive tools of sorption capacity were provided for future scale-up studies on CO_2_ capture by PSA in packed-beds of waste-derived biochar under plausible industrial conditions. Future developments and practical considerations for scaling-up PSA using biochar should involve the use of sorbent material in an industrially formed shape (e.g., pellets) to evaluate the influence of transport phenomena and temperature variations during scaled-up PSA, typically negligible at the laboratory scale by using sufficiently fine particles. The future design of continuous PSA industrial plants must be based on multicolumn systems, with each column sequentially undergoing all the operating PSA process steps (e.g., adsorption, blowdown, purge, pressurization, and any equalization steps to optimize energy consumption).

## Supplementary Material



## Notes

Procedure for biochar activation (Figure S1); PSA manual procedure for CO_2_ capture tests (Section 2); (a) median particle diameter *d*(0.5); (b) Sauter mean diameter *d*(3,2) (Figure S2); CO_2_ response curves as CO_2_ outlet molar flow rates, for *BC0.15act* (*PSAn*, *n*th *PSA* adsorption cycle with *n* = 1,..., 5) and blank tests at *P* = 5 bar_a_ (a), 7 bar_a_ (b), 9 bar_a_ (c) (Figure S3); experimental CO_2_ sorption capacities (*Y*_exp_) as functions of pressure (*P*) (Table S1); δ plot (eq 3) as a function of progressive number of experiments (Figure S4); φ plot (eq 6) as a function of pressure *P* (bar_a_) (Figure S5); and EDS map of element distribution in *BC0.15ar*, *BC0.15act, BC0.30ar* and *BC0.30act* (Figures S6–S9) (PDF)

## Nomenclature

**Symbols**
*c*_CO2,out_: Instantaneous outlet CO_2_ concentration
*ER*: Equivalence ratios
*K*: Equilibrium constant of CO_2_ desorption (Langmuir isotherm)
*P*: Operative Pressure
*p*_CO2_: CO_2_ partial pressure
*S*_BET_: Specific surface area
*V*_BJH_: Cumulative desorption pores volume
*Y*: CO_2_-sorption capacity
*Y*′: CO_2_-sorption capacity from empirical linear regression model
*Y*″: CO_2_-sorption capacity from Langmuir isotherm
*Y*_exp_: Experimental CO_2_-sorption capacity
*Y*_MAX_: Maximum asymptotic CO_2_-sorption capacity
β_*i*_: *Y*′ regression model coefficients
δ: Deviation parameter between *Y*_exp_ and *Y*′
φ: Deviation parameter between *Y*′ and *Y*″
**Abbreviations**
A–C: Active Column
*act*: Activated
*ar*: As-received
BET: Brunauer-Emmet-Teller
BJH: Barrett-Joiner-Halenda
BSE: BackScattered Electrons
DoE: Design of Experiments
EDS: Energy Dispersive X-ray Spectroscopy
FOWDTM: First Order With Dead Time Model
PSA: Pressure Swing Adsorption
PSA*n*: *n*th replication of a PSA run
PSD: Particle Size Distribution
SEM: Scanning Electron Microscopy
V: Valve
