# Supplementary material for: Biochar from Waste Vineyard Pruning as a CO2 Sorbent in Pressure Swing Adsorption: Experimental and Modeling Study
Source: ACS Omega. 2025 Jun 11;10(24):25394–404. doi: 10.1021/acsomega.5c00513 (PMC12199031; doi:10.1021/acsomega.5c00513)
Supplement: Supplementary file 1 [file ao5c00513_si_001.pdf]

SUPPLEMENTARY INFORMATION of

Biochar from Waste Vineyard Pruning as a CO_2 Sorbent in Pressure Swing Adsorption: Experimental and Modelling Study

*Daniel Mammarella [a] *, Katia Gallucci [a], Andrea Di Giuliano [a]*

[a] Department of Industrial and Information Engineering and Economics - University of L'Aquila, Piazzale E. Pontieri 1, Loc. Monteluco di Roio, 67100 L'Aquila (AQ), Italy

1. Biochar activation

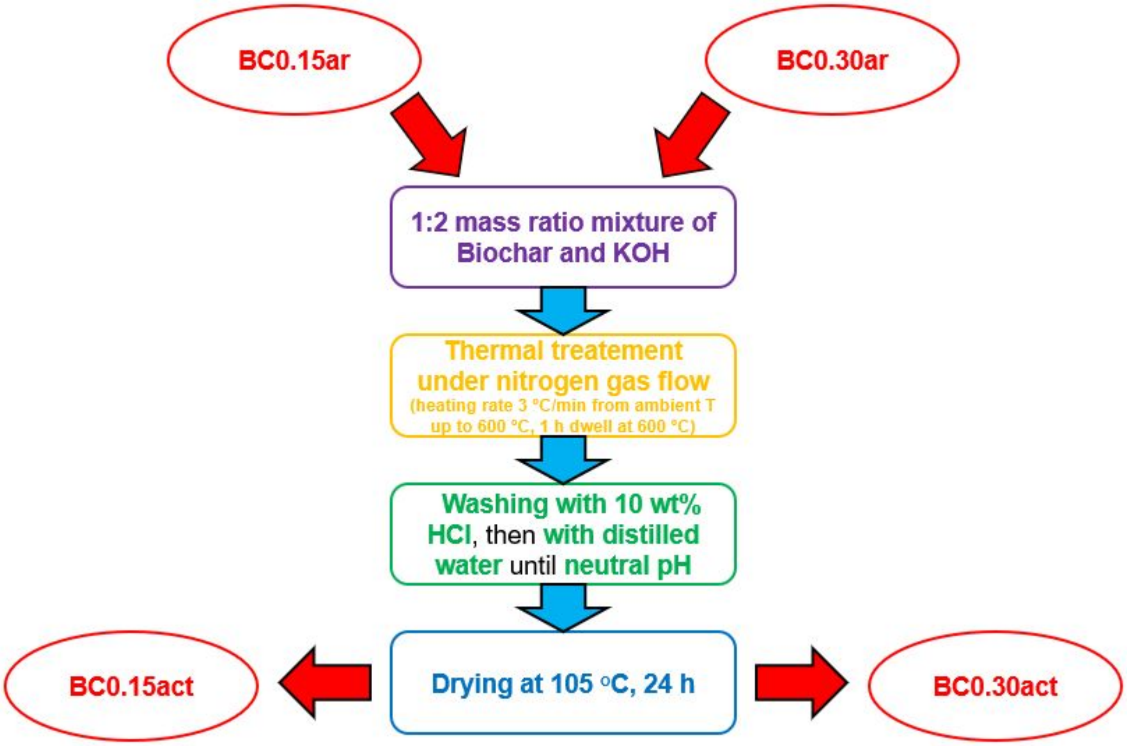

Fig. S1. Procedure for biochar activation (adapted from [1]).

2. PSA manual procedure for CO_2 capture tests

The manual valves management for PSA tests is described in the following, with reference to Fig.1 of the main manuscript. The V-D valve was always open during all tests to ensure N_2 dilution to the analyser. During the adsorption phase, valves V-A, V-B were open, V-E and V-H directed to feed CO_2/N_2 mixture downwards into A-C. For the regeneration phase, V-A and V-G were closed, V-H was open to vent for rapid depressurization while V-E was switched to send a maximum flow-rate of N_2 upwards in A-C. At the end of the regeneration step, the valves are repositioned as per the operation of a new adsorption phase.

3. Additional experimental data on CO_2 Adsorption tests by biochar

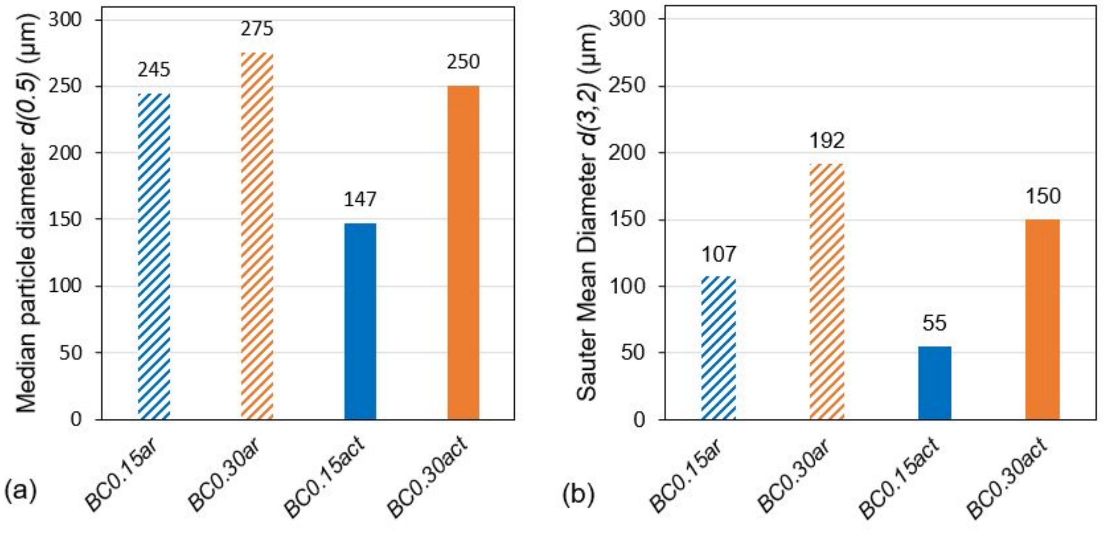

Fig. S2. (a) Median particle diameter *d(0.5)*; (b) Sauter Mean Diameter *d(3,2)*.

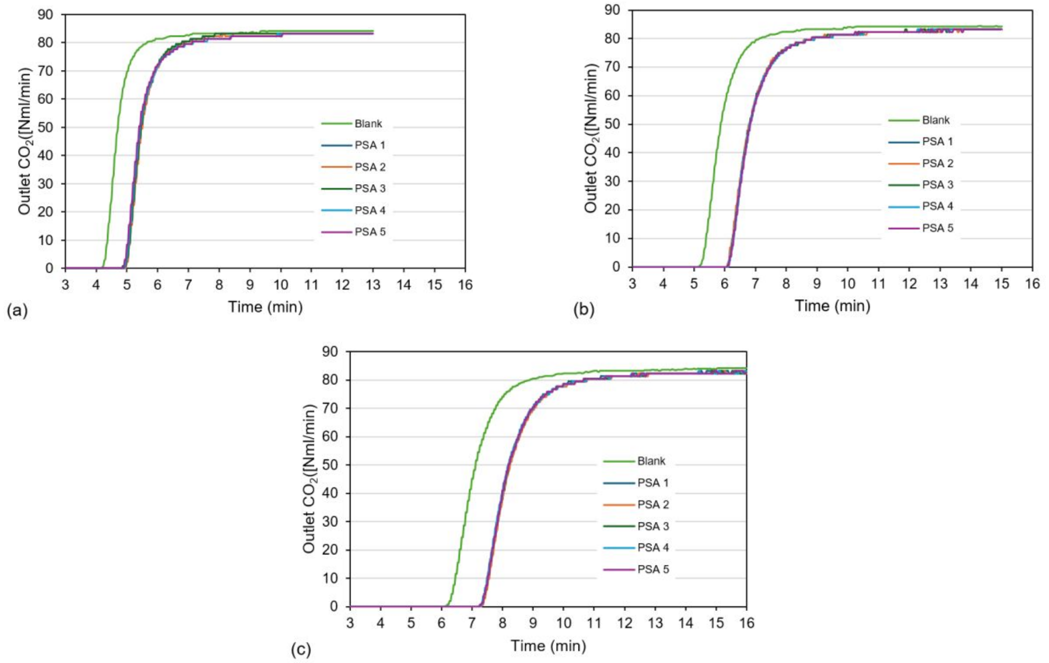

Fig. S3. CO_2 response curves as CO_2 outlet molar flow rates, for *BC0.15act* (PSA*n*, n^{th} PSA adsorption cycle with *n*=1, …, 5) and blank tests at P = 5 bar_a (a), 7 bar_a (b), 9 bar_a (c).

Table S1. Experimental CO_2 sorption capacities (Y_{exp}) as functions of pressure (P), expressed as 95% confidence intervals out of the five repetitions.

P	Y_{exp} ($mmol_{CO2}/g_{sorbent}$)			
(bar_a)	*BC0.15ar*	*BC0.30ar*	*BC0.15act*	*BC0.30act*
5	2.52 ± 0.17	2.05 ± 0.03	3.99 ± 0.10	3.51 ± 0.16
7	3.01 ± 0.05	2.61 ± 0.08	4.94 ± 0.07	4.24 ± 0.07
9	3.46 ± 0.12	3.14 ± 0.18	5.73 ± 0.12	5.00 ± 0.07

4. Additional experimental data on CO_2 Adsorption modelling

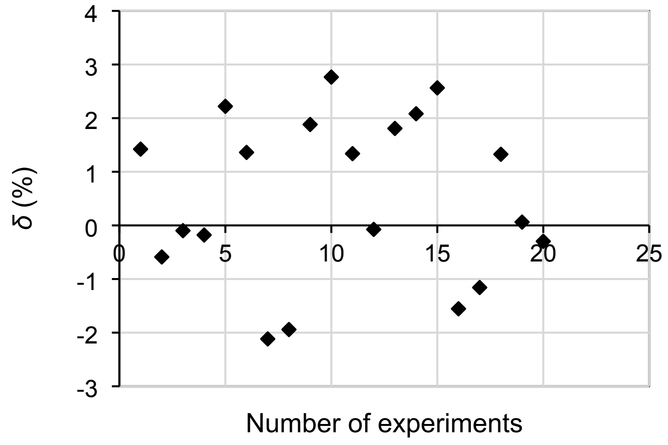

Fig. S4. δ plot (Equation (3)) as a function of progressive number of experiments.

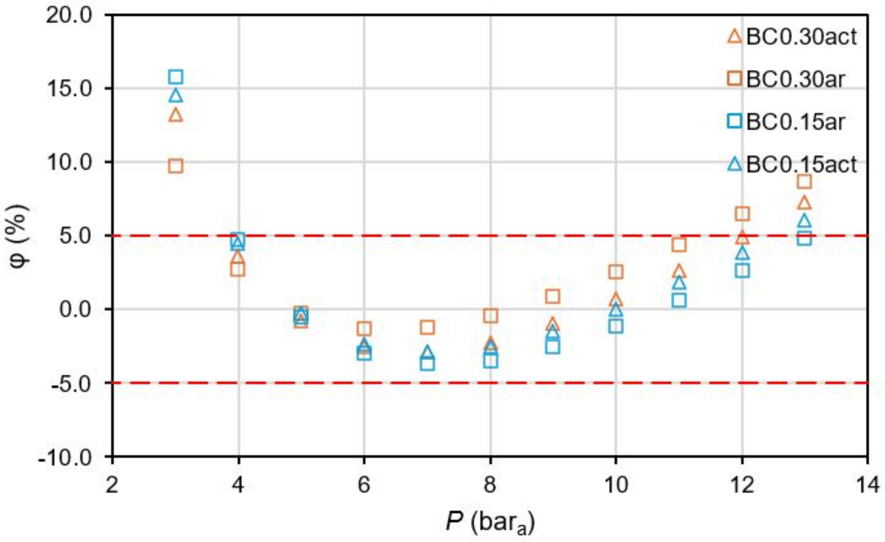

Fig. S5. φ plot (Equation (6)) as a function of pressure P (bar$_a$).

5. SEM-EDS single-element maps

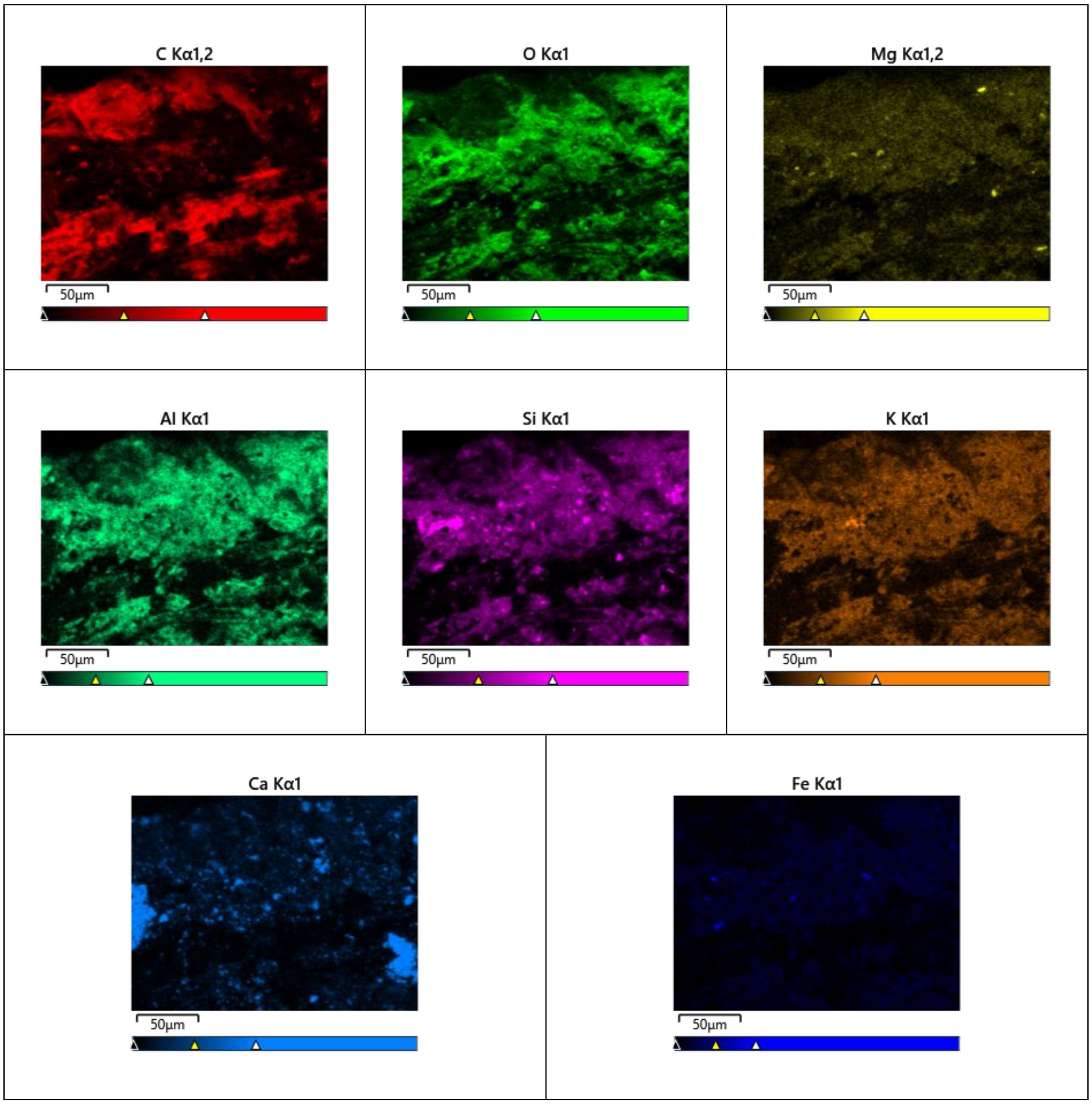

Fig. S6. SEM-EDS map of element distribution in *BC0.15ar*.

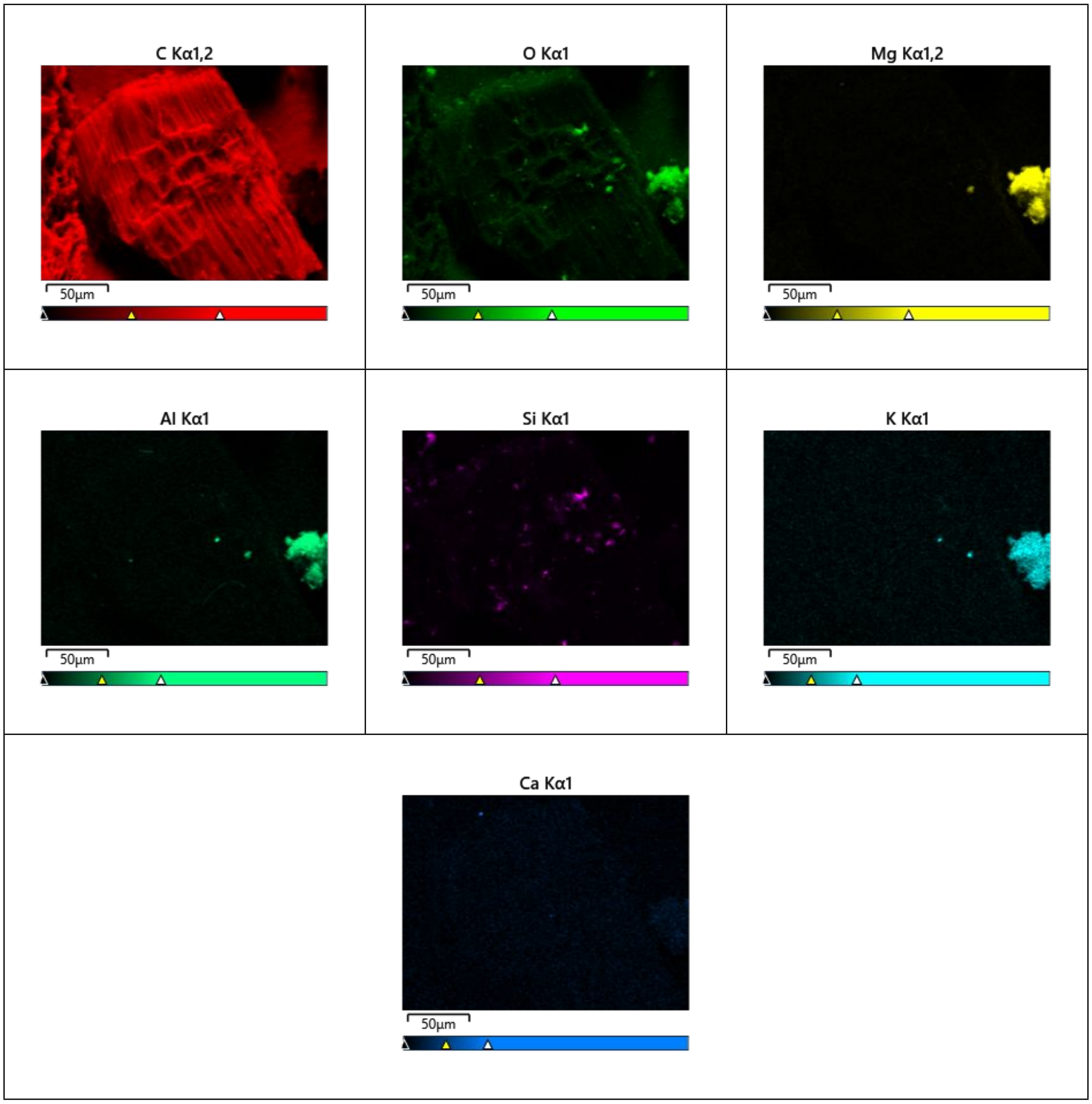

Fig. S7. SEM-EDS map of element distribution in *BC0.15act*.

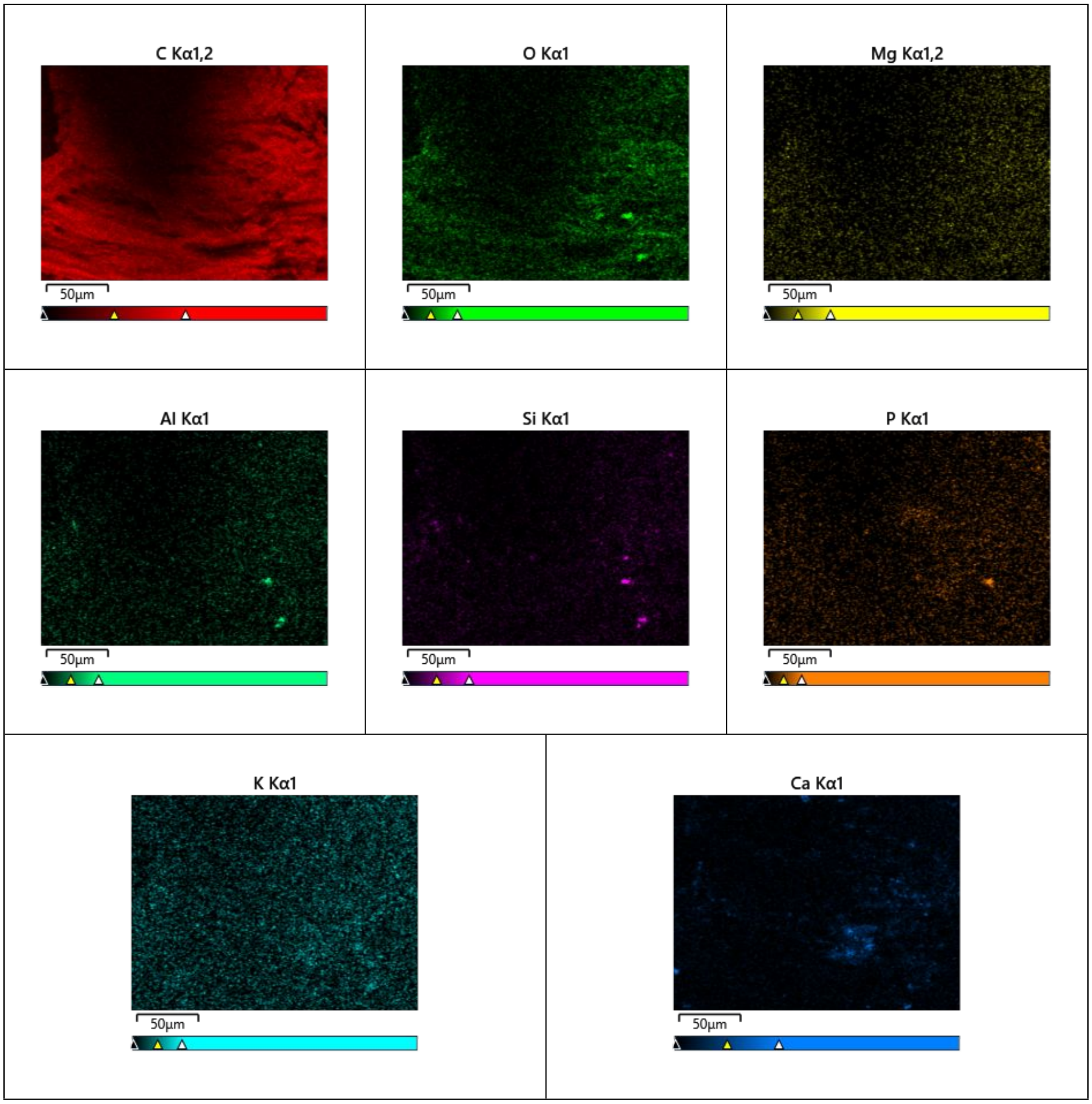

Fig. S8. SEM-EDS map of element distribution in *BC0.30ar*.

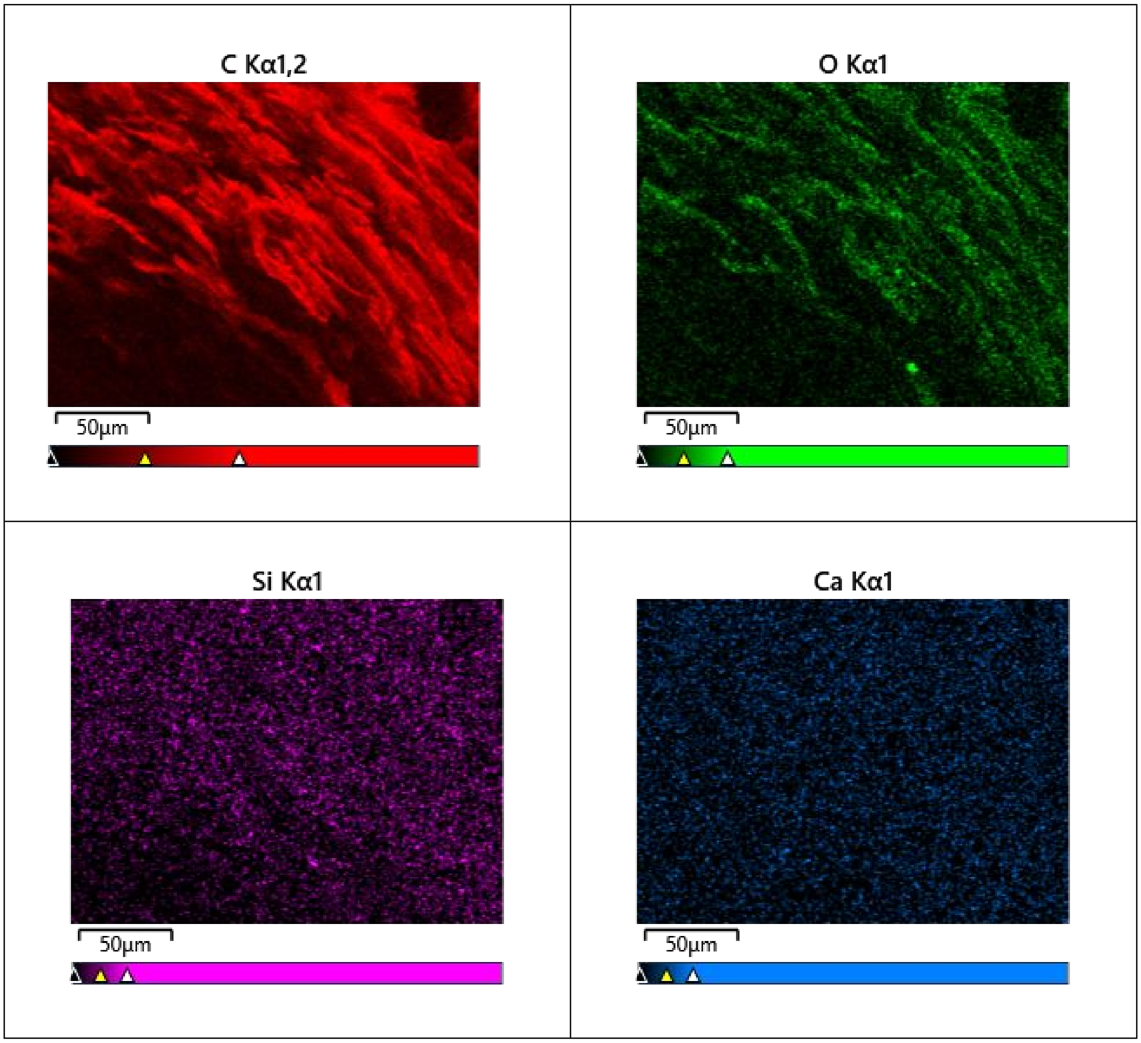

Fig. S9. SEM-EDS map of element distribution in *BC0.30act*.

References

(1) Gallucci, K.; Taglieri, L.; Papa, A. A.; Di Lauro, F.; Ahmad, Z.; Gallifuoco, A. Non-Energy Valorization of Residual Biomasses via HTC: CO2 Capture onto Activated Hydrochars. *Applied Sciences (Switzerland)* **2020**, *10* (5). https://doi.org/10.3390/app10051879.
